# 1-(2-Methyl­benzo­yl)-3-*m*-tolyl­thio­urea

**DOI:** 10.1107/S1600536808012300

**Published:** 2008-06-07

**Authors:** B. M. Yamin, S. Yousuf, M. S. M. Yusof, T. N. D. T. Zakaria

**Affiliations:** a School of Chemical Sciences and Food Technology, Universiti Kebangsaan Malaysia, UKM 43500 Bangi Selangor, Malaysia; b HEJ Research Institute of Chemistry, International Center for Chemical and Biological Sciences, University of Karachi, Karachi 75270, Pakistan; c Department of Chemistry, Universiti Malaysia Terengganu, Manngabang Telipot, Terengganu, Malaysia

## Abstract

The molecule of the title compound, C_16_H_16_N_2_OS, is not planar; the two aromatic rings are inclined to one another by 37.59 (9)°. There are intra­molecular hydrogen bonds between the benzoyl O atom and the H atom of the thio­amide N atom, and between the thio­urea S atom and the H atom of the tolyl group. These hydrogen bonds stabilize the mol­ecule in such a way that the thio­urea group adopts a *trans*–*cis* geometry. In the crystal structure, mol­ecules are linked by N—H⋯S inter­molecular hydrogen bonds, forming centrosymmetric dimers.

## Related literature

For the crystal structure of 1-(2,3-dimethyl­phen­yl)-3-(2-methyl­benzo­yl)thio­urea, see: Khawar Rauf *et al.* (2007 ▶). For bond-length data, see: Allen *et al.* (1987 ▶).
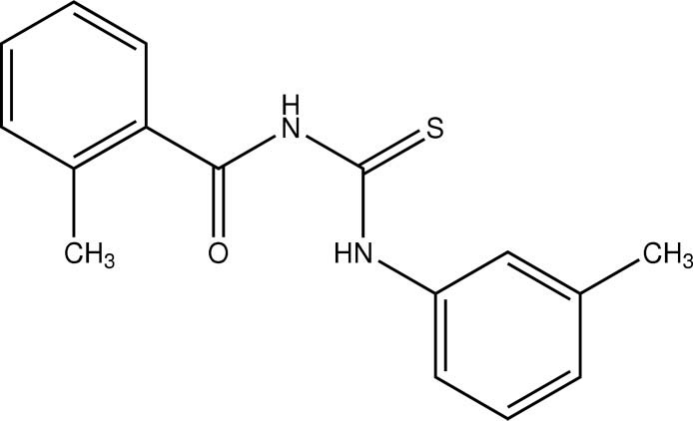


## Experimental

### 

#### Crystal data

C_16_H_16_N_2_OS
*M* *_r_* = 284.37
Triclinic, 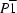

*a* = 6.440 (3) Å
*b* = 10.201 (5) Å
*c* = 11.415 (5) Å
α = 77.310 (7)°
β = 89.896 (8)°
γ = 86.468 (8)°
*V* = 730.1 (6) Å^3^
*Z* = 2
Mo *K*α radiation
μ = 0.22 mm^−1^
*T* = 298 (2) K
0.35 × 0.34 × 0.22 mm

#### Data collection

Bruker SMART APEX CCD area-detector diffractometer
Absorption correction: multi-scan (*SADABS*; Bruker, 2000 ▶) *T*_min_ = 0.927, *T*_max_ = 0.954
7240 measured reflections
2703 independent reflections
2202 reflections with *I* > 2σ(*I*)
*R*_int_ = 0.023

#### Refinement

*R*[*F* ^2^ > 2σ(*F* ^2^)] = 0.043
*wR*(*F* ^2^) = 0.125
*S* = 1.02
2703 reflections
181 parameters
H-atom parameters constrained
Δρ_max_ = 0.23 e Å^−3^
Δρ_min_ = −0.28 e Å^−3^

### 

Data collection: *SMART* (Bruker, 2000 ▶); cell refinement: *SAINT* (Bruker, 2000 ▶); data reduction: *SAINT*; program(s) used to solve structure: *SHELXS97* (Sheldrick, 2008 ▶); program(s) used to refine structure: *SHELXL97* (Sheldrick, 2008 ▶); molecular graphics: *SHELXTL* (Sheldrick, 2008 ▶); software used to prepare material for publication: *SHELXTL*, *PARST* (Nardelli, 1995 ▶) and *PLATON* (Spek, 2003 ▶).

## Supplementary Material

Crystal structure: contains datablocks global, I. DOI: 10.1107/S1600536808012300/su2052sup1.cif

Structure factors: contains datablocks I. DOI: 10.1107/S1600536808012300/su2052Isup2.hkl

Additional supplementary materials:  crystallographic information; 3D view; checkCIF report

## Figures and Tables

**Table 1:** Hydrogen-bond geometry (Å, °)

*D*—H⋯*A*	*D*—H	H⋯*A*	*D*⋯*A*	*D*—H⋯*A*
N1—H1*A*⋯S1^i^	0.86	2.74	3.407 (2)	136
N2—H2*A*⋯O1	0.86	1.97	2.658 (2)	136
C7—H7*C*⋯O1	0.96	2.52	2.933 (3)	106
C15—H15*A*⋯S1	0.93	2.54	3.168 (3)	125

Symmetry code: (i) 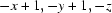.

**Fig. 1.**
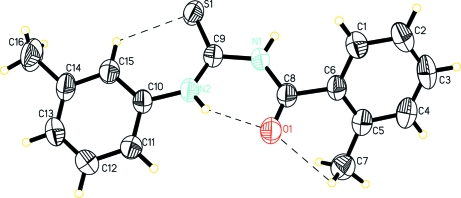
The molecular structure of compound (1), with displacement ellipsoids drawn at 50% probability level (The dashed lines indicate the intramolecular hydrogen bonds).

**Fig. 2.**
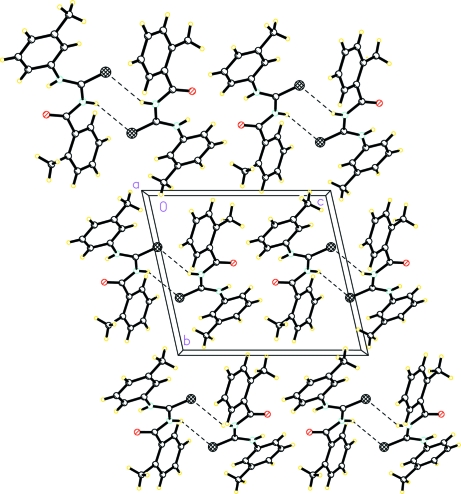
The crystal packing diagram of compound (1), showing the formation of the N-H···S hydrogen bonded dimers (Hydrogen bonds are shown by dashed lines).

## supplementary crystallographic information

### Comment

The title compound, (I), is analogus to 1-(2,3-Dimethylphenyl)-3-(2-methylbenzoyl)thiourea (II) (Khawar Rauf *et al.*, 2007), but with the 2,3-dimethyl phenyl group replaced by a 2-methyl phenyl (m-tolyl) group (Fig. 1). The molecule maintains the *trans*-*cis* configuration with respect to the position of the methyl benzoyl and 3-methyl benzene groups, respectively, relative to the thiono S1 atom. The bond lengths and angles are in normal ranges (Allen *et al.*, 1987). The central thiourea moiety, S1/N1/N2/C9, the 2-methylbenzoyl ring, (C1—C8), and the m-tolyl group (C10—C15,C16) are all relatively planar, with a maximum deviation from any best mean plane of 0.015 (2) Å for atom C10. The central thiourea moiety makes dihedral angles with the 2-methylbenzoyl and m-tolyl fragments of 49.61 (7) and 17.87 (9)°, respectively. The *trans*-*cis* geometry of the thiourea moiety is stabilized by N2—H2A···O1, C7—H7C···O1 and C15—H15A···S1 intramolecular hydrogen bonds (Table 1).

In the crystal structure of (I), symmetry related molecules are linked by the N1—H1A···S1 intermolecular hydrogen bonds (Table 1) to form cenntrosymmetric dimers (Fig 2).

### Experimental

2-methylbenzoyl chloride (9.720 g, 0.025mole) was mixed with an equimolar amount of ammonium thiocyanate (1.903 g, 0.025 mol) and 3-methyl aniline (2.701 g, 0.025 mol) in 45 ml dry acetone. The mixture was refluxed with stirring for 4 h. The solution was then filtered and left to evaporate at room temperature. Colourless crystals, suitable for X-ray aanalysis, were obtained after a few days (Yield 85%).

### Refinement

NH and C-bound H atoms were positioned geometrically and constrained to ride on their parent atoms: N—H = 0.86 and C—H = 0.93 - 0.96 Å, with *U*_iso_(H)= 1.2*U*_eq_(CH and NH), and 1.5*U*_eq_(CH_3_).

### Crystal data

**Table:**

C_16_H_16_N_2_OS	*Z* = 2
*M**_r_* = 284.37	*F*_000_ = 300
Triclinic, *P*1	*D*_x_ = 1.293 Mg m^−^^3^
Hall symbol: -P 1	Mo *K*α radiation λ = 0.71073 Å
*a* = 6.440 (3) Å	Cell parameters from 2987 reflections
*b* = 10.201 (5) Å	θ = 1.8–25.5º
*c* = 11.415 (5) Å	µ = 0.22 mm^−^^1^
α = 77.310 (7)º	*T* = 298 (2) K
β = 89.896 (8)º	Block, colorless
γ = 86.468 (8)º	0.35 × 0.34 × 0.22 mm
*V* = 730.1 (6) Å^3^	

### Data collection

**Table:**

Bruker SMART APEX CCD area-detector diffractometer	2703 independent reflections
Radiation source: fine-focus sealed tube	2202 reflections with *I* > 2σ(*I*)
Monochromator: graphite	*R*_int_ = 0.023
Detector resolution: 83.66 pixels mm^-1^	θ_max_ = 25.5º
*T* = 298(2) K	θ_min_ = 1.8º
ω scans	*h* = −7→7
Absorption correction: multi-scan(SADABS; Bruker, 2000)	*k* = −12→12
*T*_min_ = 0.927, *T*_max_ = 0.954	*l* = −13→13
7240 measured reflections	

### Refinement

**Table:**

Refinement on *F*^2^	Secondary atom site location: difference Fourier map
Least-squares matrix: full	Hydrogen site location: inferred from neighbouring sites
*R*[*F*^2^ > 2σ(*F*^2^)] = 0.043	H-atom parameters constrained
*wR*(*F*^2^) = 0.125	*w* = 1/[σ^2^(*F*_o_^2^) + (0.077*P*)^2^ + 0.124*P*] where *P* = (*F*_o_^2^ + 2*F*_c_^2^)/3
*S* = 1.02	(Δ/σ)_max_ < 0.000
2703 reflections	Δρ_max_ = 0.23 e Å^−^^3^
181 parameters	Δρ_min_ = −0.28 e Å^−^^3^
Primary atom site location: structure-invariant direct methods	Extinction correction: none

### Special details

**Table:**

Geometry. Bond distances, angles etc. have been calculated using the rounded fractional coordinates. All su's are estimated from the variances of the (full) variance-covariance matrix. The cell esds are taken into account in the estimation of distances, angles and torsion angles
Refinement. Refinement of *F*^2^ against ALL reflections. The weighted *R*-factor *wR* and goodness of fit *S* are based on *F*^2^, conventional *R*-factors *R* are based on *F*, with *F* set to zero for negative *F*^2^. The threshold expression of *F*^2^ > σ(*F*^2^) is used only for calculating *R*-factors(gt) *etc*. and is not relevant to the choice of reflections for refinement. *R*-factors based on *F*^2^ are statistically about twice as large as those based on *F*, and *R*- factors based on ALL data will be even larger.

### Fractional atomic coordinates and isotropic or equivalent isotropic displacement parameters (Å^2^)

**Table:**

	*x*	*y*	*z*	*U*_iso_*/*U*_eq_	
S1	0.32952 (9)	0.66913 (5)	0.02660 (4)	0.0657 (2)	
O1	0.5770 (2)	0.43595 (14)	0.39009 (11)	0.0589 (4)	
N1	0.5747 (2)	0.51255 (14)	0.18710 (13)	0.0474 (5)	
N2	0.3091 (2)	0.62497 (14)	0.26753 (12)	0.0463 (4)	
C1	0.9854 (3)	0.3839 (2)	0.19650 (18)	0.0549 (6)	
C2	1.1512 (3)	0.2991 (2)	0.1779 (2)	0.0663 (8)	
C3	1.1594 (3)	0.1659 (2)	0.2386 (2)	0.0708 (8)	
C4	1.0059 (3)	0.1190 (2)	0.3173 (2)	0.0632 (7)	
C5	0.8385 (3)	0.20259 (18)	0.33940 (17)	0.0502 (6)	
C6	0.8294 (2)	0.33688 (17)	0.27600 (15)	0.0450 (5)	
C7	0.6744 (3)	0.1451 (2)	0.4262 (2)	0.0664 (7)	
C8	0.6511 (3)	0.43112 (17)	0.29297 (15)	0.0445 (5)	
C9	0.4011 (3)	0.60340 (16)	0.16802 (15)	0.0445 (5)	
C10	0.1164 (2)	0.69333 (16)	0.28323 (15)	0.0421 (5)	
C11	0.0350 (3)	0.65998 (18)	0.39738 (16)	0.0508 (6)	
C12	−0.1571 (3)	0.7154 (2)	0.42026 (19)	0.0611 (7)	
C13	−0.2680 (3)	0.80384 (19)	0.33001 (19)	0.0568 (7)	
C14	−0.1870 (3)	0.84061 (19)	0.21718 (18)	0.0560 (6)	
C15	0.0074 (3)	0.78556 (19)	0.19379 (16)	0.0551 (6)	
C16	−0.3049 (4)	0.9400 (3)	0.1191 (2)	0.0903 (10)	
H1A	0.64410	0.50630	0.12390	0.0570*	
H1B	0.97800	0.47350	0.15540	0.0660*	
H2A	0.37820	0.59190	0.33290	0.0560*	
H2B	1.25610	0.33120	0.12520	0.0800*	
H3A	1.26980	0.10770	0.22600	0.0850*	
H4A	1.01400	0.02880	0.35700	0.0760*	
H7A	0.71130	0.05180	0.45960	0.1000*	
H7B	0.54280	0.15370	0.38500	0.1000*	
H7C	0.66430	0.19330	0.48970	0.1000*	
H11A	0.10970	0.60040	0.45840	0.0610*	
H12A	−0.21230	0.69310	0.49690	0.0730*	
H13A	−0.39920	0.83900	0.34580	0.0680*	
H15A	0.06450	0.81070	0.11790	0.0660*	
H16A	−0.43520	0.96820	0.14950	0.1350*	
H16B	−0.33020	0.89820	0.05330	0.1350*	
H16C	−0.22440	1.01680	0.09180	0.1350*	

### Atomic displacement parameters (Å^2^)

**Table:**

	*U*^11^	*U*^22^	*U*^33^	*U*^12^	*U*^13^	*U*^23^
S1	0.0808 (4)	0.0649 (3)	0.0427 (3)	0.0316 (3)	0.0035 (2)	−0.0031 (2)
O1	0.0592 (8)	0.0699 (8)	0.0445 (7)	0.0245 (6)	−0.0033 (6)	−0.0134 (6)
N1	0.0440 (8)	0.0508 (8)	0.0439 (8)	0.0123 (6)	0.0053 (6)	−0.0071 (6)
N2	0.0435 (8)	0.0519 (8)	0.0413 (7)	0.0144 (6)	−0.0033 (6)	−0.0100 (6)
C1	0.0418 (10)	0.0589 (11)	0.0643 (12)	0.0012 (8)	0.0004 (8)	−0.0155 (9)
C2	0.0393 (10)	0.0854 (15)	0.0775 (14)	0.0030 (10)	0.0073 (9)	−0.0269 (12)
C3	0.0463 (11)	0.0783 (15)	0.0936 (16)	0.0214 (10)	−0.0050 (11)	−0.0382 (13)
C4	0.0573 (12)	0.0524 (11)	0.0802 (14)	0.0146 (9)	−0.0122 (10)	−0.0202 (10)
C5	0.0450 (9)	0.0498 (10)	0.0570 (10)	0.0062 (8)	−0.0082 (8)	−0.0165 (8)
C6	0.0366 (9)	0.0512 (9)	0.0484 (9)	0.0063 (7)	−0.0059 (7)	−0.0159 (8)
C7	0.0646 (13)	0.0550 (11)	0.0743 (14)	0.0019 (10)	0.0050 (11)	−0.0044 (10)
C8	0.0399 (9)	0.0463 (9)	0.0469 (9)	0.0048 (7)	−0.0028 (7)	−0.0116 (7)
C9	0.0420 (9)	0.0400 (8)	0.0494 (10)	0.0053 (7)	0.0013 (7)	−0.0078 (7)
C10	0.0406 (9)	0.0409 (8)	0.0456 (9)	0.0055 (7)	−0.0024 (7)	−0.0134 (7)
C11	0.0534 (10)	0.0515 (10)	0.0448 (9)	0.0085 (8)	0.0006 (8)	−0.0082 (7)
C12	0.0589 (12)	0.0628 (12)	0.0598 (12)	0.0062 (9)	0.0156 (9)	−0.0122 (9)
C13	0.0421 (10)	0.0567 (11)	0.0730 (13)	0.0097 (8)	0.0028 (9)	−0.0211 (9)
C14	0.0534 (11)	0.0524 (10)	0.0614 (12)	0.0180 (8)	−0.0097 (9)	−0.0163 (9)
C15	0.0590 (11)	0.0556 (10)	0.0462 (10)	0.0176 (9)	0.0015 (8)	−0.0071 (8)
C16	0.0882 (17)	0.0929 (17)	0.0776 (16)	0.0491 (14)	−0.0173 (13)	−0.0073 (13)

### Geometric parameters (Å, °)

**Table:**

S1—C9	1.6605 (19)	C12—C13	1.379 (3)
O1—C8	1.216 (2)	C13—C14	1.372 (3)
N1—C8	1.380 (2)	C14—C16	1.506 (3)
N1—C9	1.393 (2)	C14—C15	1.390 (3)
N2—C9	1.335 (2)	C1—H1B	0.9300
N2—C10	1.416 (2)	C2—H2B	0.9300
N1—H1A	0.8600	C3—H3A	0.9300
N2—H2A	0.8600	C4—H4A	0.9300
C1—C6	1.388 (3)	C7—H7A	0.9600
C1—C2	1.378 (3)	C7—H7B	0.9600
C2—C3	1.381 (3)	C7—H7C	0.9600
C3—C4	1.371 (3)	C11—H11A	0.9300
C4—C5	1.390 (3)	C12—H12A	0.9300
C5—C6	1.400 (3)	C13—H13A	0.9300
C5—C7	1.503 (3)	C15—H15A	0.9300
C6—C8	1.492 (3)	C16—H16A	0.9600
C10—C15	1.386 (3)	C16—H16B	0.9600
C10—C11	1.383 (3)	C16—H16C	0.9600
C11—C12	1.377 (3)		
			
C8—N1—C9	129.20 (15)	C13—C14—C15	119.23 (18)
C9—N2—C10	130.59 (14)	C10—C15—C14	120.21 (17)
C8—N1—H1A	115.00	C2—C1—H1B	120.00
C9—N1—H1A	115.00	C6—C1—H1B	120.00
C10—N2—H2A	115.00	C1—C2—H2B	120.00
C9—N2—H2A	115.00	C3—C2—H2B	120.00
C2—C1—C6	120.68 (19)	C2—C3—H3A	120.00
C1—C2—C3	119.10 (19)	C4—C3—H3A	120.00
C2—C3—C4	120.45 (19)	C3—C4—H4A	119.00
C3—C4—C5	121.78 (19)	C5—C4—H4A	119.00
C4—C5—C6	117.43 (17)	C5—C7—H7A	110.00
C4—C5—C7	119.31 (17)	C5—C7—H7B	110.00
C6—C5—C7	123.23 (17)	C5—C7—H7C	109.00
C1—C6—C8	119.24 (16)	H7A—C7—H7B	110.00
C5—C6—C8	120.22 (15)	H7A—C7—H7C	109.00
C1—C6—C5	120.54 (16)	H7B—C7—H7C	109.00
O1—C8—N1	122.48 (17)	C10—C11—H11A	120.00
O1—C8—C6	123.92 (16)	C12—C11—H11A	120.00
N1—C8—C6	113.60 (14)	C11—C12—H12A	120.00
N1—C9—N2	115.10 (15)	C13—C12—H12A	120.00
S1—C9—N1	117.23 (13)	C12—C13—H13A	120.00
S1—C9—N2	127.65 (14)	C14—C13—H13A	120.00
N2—C10—C15	124.98 (15)	C10—C15—H15A	120.00
C11—C10—C15	119.83 (15)	C14—C15—H15A	120.00
N2—C10—C11	115.18 (15)	C14—C16—H16A	109.00
C10—C11—C12	119.69 (17)	C14—C16—H16B	109.00
C11—C12—C13	120.27 (19)	C14—C16—H16C	109.00
C12—C13—C14	120.72 (18)	H16A—C16—H16B	109.00
C13—C14—C16	120.97 (18)	H16A—C16—H16C	110.00
C15—C14—C16	119.80 (18)	H16B—C16—H16C	109.00
			
C9—N1—C8—O1	−5.0 (3)	C7—C5—C6—C1	−179.65 (18)
C9—N1—C8—C6	174.26 (16)	C7—C5—C6—C8	0.2 (3)
C8—N1—C9—S1	−170.78 (15)	C1—C6—C8—O1	−137.6 (2)
C8—N1—C9—N2	7.9 (3)	C1—C6—C8—N1	43.1 (2)
C10—N2—C9—S1	8.9 (3)	C5—C6—C8—O1	42.6 (3)
C10—N2—C9—N1	−169.59 (16)	C5—C6—C8—N1	−136.71 (17)
C9—N2—C10—C11	159.19 (18)	N2—C10—C11—C12	−176.51 (17)
C9—N2—C10—C15	−19.4 (3)	C15—C10—C11—C12	2.1 (3)
C6—C1—C2—C3	0.6 (3)	N2—C10—C15—C14	175.98 (17)
C2—C1—C6—C5	0.5 (3)	C11—C10—C15—C14	−2.5 (3)
C2—C1—C6—C8	−179.37 (18)	C10—C11—C12—C13	−0.1 (3)
C1—C2—C3—C4	−0.7 (3)	C11—C12—C13—C14	−1.7 (3)
C2—C3—C4—C5	−0.3 (3)	C12—C13—C14—C15	1.3 (3)
C3—C4—C5—C6	1.4 (3)	C12—C13—C14—C16	−178.4 (2)
C3—C4—C5—C7	179.67 (19)	C13—C14—C15—C10	0.8 (3)
C4—C5—C6—C1	−1.5 (3)	C16—C14—C15—C10	−179.6 (2)
C4—C5—C6—C8	178.38 (17)		

### Hydrogen-bond geometry (Å, °)

**Table:**

*D*—H···*A*	*D*—H	H···*A*	*D*···*A*	*D*—H···*A*
N1—H1A···S1^i^	0.86	2.74	3.407 (2)	136
N2—H2A···O1	0.86	1.97	2.658 (2)	136
C7—H7C···O1	0.96	2.52	2.933 (3)	106
C15—H15A···S1	0.93	2.54	3.168 (3)	125

Symmetry codes: (i) −*x*+1, −*y*+1, −*z*.
